# Assessing Analytical Performance and Correct Classification for Cardiac Troponin Deltas Across Diagnostic Pathways Used for Myocardial Infarction

**DOI:** 10.3390/diagnostics15131652

**Published:** 2025-06-28

**Authors:** Peter A. Kavsak, Sameer Sharif, Wael L. Demian, Won-Shik Choi, Emilie P. Belley-Cote, Jennifer Taher, Jennifer L. Shea, David W. Blank, Michael Knauer, Laurel Thorlacius, Joshua E. Raizman, Yun Huang, Daniel R. Beriault, Angela W. S. Fung, Paul M. Yip, Lorna Clark, Beth L. Abramson, Steven M. Friedman, Jesse McLaren, Paul Atkinson, Annabel Chen-Tournoux, Neville Suskin, Marco L. A. Sivilotti, Venkatesh Thiruganasambandamoorthy, Frank Scheuermeyer, Karin H. Humphries, Kristin M. Aakre, Shawn E. Mondoux, Craig Ainsworth, Flavia Borges, Andrew Worster, Andrew McRae, Allan S. Jaffe

**Affiliations:** 1 Faculty of Health Sciences, McMaster University, Hamilton, ON L8N 3Z5, Canada; 2 Population Health Research Institute, Hamilton, ON L8L 2X2, Canada; 3 University of Toronto, Toronto, ON M5T 1R8, Canadabeth.abramson@unityhealth.to (B.L.A.);; 4 Department of Laboratory Medicine, Saint John Regional Hospital, Saint John, NB E2L 4L2, Canada; 5 McGill University, Montréal, QC H3A 0G4, Canada; david.blank.med@ssss.gouv.qc.ca (D.W.B.);; 6 Western University, London, ON N6A 5B9, Canada; 7 University of Manitoba, Winnipeg, MB R2H 2A6, Canada; lthorlacius@sharedhealthmb.ca; 8 University of Alberta, Edmonton, AB T6G 2G4, Canada; 9 Queen’s University, Kingston, ON KL7 3N6, Canada; yun.huang@kingstonhsc.ca (Y.H.);; 10 University of British Columbia, Vancouver, BC V6S 1Z2, Canada; afung7@providencehealth.bc.ca (A.W.S.F.);; 11 Dalhousie Medicine, Saint John, NB E2K 5E2, Canada; 12 University of Ottawa, Ottawa, ON K1N 6N5, Canada; 13 Department of Medical Biochemistry and Pharmacology & Department of Heart Disease, University Hospital, Haukeland, 5007 Bergen, Norway; 14 Department of Clinical Science, University of Bergen, 5021 Bergen, Norway; 15 University of Calgary, Calgary, AB T2N1N4, Canada; 16 Mayo Clinic and Medical Center, Rochester, MN 55905, USA

**Keywords:** cardiac troponin delta, high-sensitivity cardiac troponin, diagnostic pathways, European Society of Cardiology (ESC), High-STEACS, Common Change Criteria (3C), misclassification, analytical variation

## Abstract

**Background:** In the emergency setting, many diagnostic pathways incorporate change in high-sensitivity cardiac troponin (hs-cTn) concentrations (i.e., the delta) to classify patients as low-risk (rule-out) or high-risk (rule-in) for possible myocardial infarction (MI). However, the impact of analytical variation on the delta for correct classification is unknown, especially at concentrations below and around the 99th percentile. Our objective was to assess the impact of delta variation for correct risk classification across the European Society of Cardiology (ESC 0/1 h and 0/2 h), the High-STEACS, and the common change criteria (3C) pathways. **Methods**: A yearlong accuracy study for hs-cTnT was performed where laboratories across Canada tested three patient-based samples (level 1 target value = 6 ng/L, level 2 target value = 9 ng/L, level 3 target value = 12 ng/L) monthly across 41 different analyzers. The assigned low-delta between levels 1 and 2 was 3 ng/L (i.e., 9 − 6 = 3 ng/L) and the assigned high-delta between levels 1 and 3 was 6 ng/L (i.e., 12 − 6 = 6 ng/L). The low- and high-deltas for each analyzer were determined monthly from the measured values, with the difference calculated from the assigned deltas. The obtained deltas were then assessed via the different pathways on correct classification (i.e., percent correct with 95% confidence intervals, CI) and using non-parametric analyses. **Results**: The median (interquartile range) difference between the measured versus assigned low-delta (n = 436) and high-delta (n = 439) was −1 ng/L (−1 to 0). The correct classification differed among the pathways. The ESC 0/1 h pathway yielded the lowest percentage of correct classification at 35.3% (95% CI: 30.8 to 40.0) for the low-delta and 90.0% (95% CI: 86.8 to 92.6) for the high-delta. The 3C and ESC 0/2 h pathways yielded higher and equivalent estimates on correct classification: 95.2% (95% CI: 92.7 to 97.0) for the low-delta and 98.2% (95% CI: 96.4 to 99.2) for the high-delta. The High-STEACS pathway yielded 99.5% (95% CI: 98.4 to 99.9) of correct classifications for the high-delta but only 36.2% (95% CI: 31.7 to 40.9) for the low-delta. **Conclusions**: Analytical variation will impact risk classification for MI when using hs-cTn deltas alone per the pathways. The 3C and ESC 0/2 h pathways have <5% misclassification when using deltas for hs-cTnT in this dataset. Additional studies with different hs-cTnI assays at concentrations below and near the 99th percentile are warranted to confirm these findings.

## 1. Introduction

The “rise/fall” criteria using high-sensitivity cardiac troponin (hs-cTn) assays or cardiac specific biomarkers is not only important for the formal diagnosis of myocardial infarction (MI) but also to help risk-stratify approaches to identify patients at high-risk (rule-in) and low-risk (rule-out) for MI [1,2,3]. However, defining an optimal delta change value to use to do this is complex. Many of the proposed change criteria were developed based on observational clinical data and may not necessarily take into account important analytical issues. To that end, in large studies, the likelihood that large numbers of patients are misclassified, leading to a clinical signal, is unlikely [4]. Indeed, many of these patients go from low-risk or high-risk to the so-called observe category. At times, the criteria and diagnoses in this group are less clear [5]. Nonetheless, this may cause confusion for clinicians.

Many published cardiac troponin-based pathways incorporate a change (i.e., the delta) in cardiac troponin concentrations between serial blood draws (i.e., time between blood draws varies from 1 to 3 h) [2,3,4,5,6,7,8]. This short time interval reflects the time-sensitive nature of cardiac ischemia and exquisite sensitivity of current assays, as well as time pressure on emergency department (ED) through-put.

Historically, for cardiac troponin the 2007 Universal Definition of Myocardial Infarction stated “recurrent infarction is diagnosed if there is a >20% increase of the value in the second sample” [9]. The 20% criterion (i.e., a relative change or delta) was based on analytical performance of the cardiac troponin assays in the early 2000s and before the advent of hs-cTn assays. Also, the time between blood sampling for cardiac troponin measurements during this period was typically 6 h apart with some reports indicating a change in cardiac troponin of >30% improving diagnostic performance [10,11]. Even with the non-hs-cTn assays, data indicated that analytical performance was suitable to further decrease the time between serial measurements down to 3 h [12]. Initially, 3 h between measurements for hs-cTn assays was also proposed, with data suggesting that deltas could be assessed both in absolute values and as well as relative values [13,14,15,16]. Yet shortening this time interval between serial testing can result in the random, analytical variation of the assay itself contributing substantially to the delta, with data indicating that at lower hs-cTn values, an absolute delta provides important diagnostic and prognostic information in and outside of the emergency setting [14,17].

There have been far more clinical than analytical evaluations of these diagnostic pathways, highlighting the absence of robust analytical criteria to evaluate the absolute analytical error for the hs-cTn assays [18]. The bias is that most true MI events provide a robust change in values, and that those without such changes can be triaged clinically or with additional hs-cTn testing. One of the first hs-cTn pathways involving this delta was by the Advantageous Predictors of Acute Coronary Syndrome Evaluation (APACE) study investigators, who used the absolute difference between 0 and 1 h (“0/1 h”) [19]. These investigators also derived a different pathway for samples 0/2 h [20]. Noted limitations of the APACE algorithms for hs-cTnT beyond this very short time interval are the use of unique cutoffs and delta values for the 0/1 h and 0/2 h pathways and for each individual hs-cTn assay. However, other pathways have proposed numerically identical cutoffs and deltas despite using different hs-cTn assays. For example, the High-STEACS (High-Sensitivity Troponin in the Evaluation of Patients With Acute Coronary Syndrome) pathway uses a delta < 3 ng/L for hs-cTn concentrations between 5 ng/L and the 99th percentile (i.e., upper reference limit, URL) for low-risk classification and with a delta ≥ 3 ng/L for high-risk classification [21,22]. Similarly, in the common change criteria (3C) pathway for an initial concentration < 10 ng/L, a delta ≤ 3 ng/L identifies low-risk, while a delta > 3 ng/L indicates high-risk [8].

It is well recognized that analytical variation near the given cutoff for any given assay will result in misclassification when using a single low hs-cTnT concentration [23,24]. However, the impact of analytical variation on patient classification when using small delta differences between repeated measurements is less well appreciated, especially for concentrations below and near the 99th percentile. Our objective was to evaluate the effect of using a low-delta (3 ng/L change) versus a high-delta (6 ng/L change) on the correct risk classification and accuracy with the hs-cTnT assay via four different cardiac troponin-based pathways (ESC 0/1 h, ESC 0/2 h, High-STEACS, 3C). Even if only a modest number of patients are misclassified, it would be important for clinicians to be aware and surveille for this group.

## 2. Materials and Methods

Details of this analytical study assessing accuracy for hs-cTn assays have been previously reported [18,25,26]. Briefly, three different human-based pools were made to test assay variation at low hs-cTn levels (level 1), near the female 99th percentile (URL) (level 2) and near the male URL (level 3). The materials were sent frozen (stored below –70 °C and sent on dry ice) to laboratories across Canada to run monthly (i.e., each month three frozen aliquots were removed from the freezer and tested for each level) from January 2022 through to December 2022, as part of a prospective trial on the impact of the female sex-specific 99th percentile [27]. To standardize the process for handling and testing the samples, the same instructions were provided to each laboratory [18,25,26]. Specifically, testing was performed mid-month (i.e., between days 10 to 20) where laboratory staff would remove frozen aliquots (volume of sample per aliquot ≥ 300 ul) from the freezer and allow the aliquots to thaw for 15 min at room temperature. After 15 min, the aliquots were mixed to confirm that the thaw process was completed and then centrifuged (3000 g) for 10 min prior to testing on the instruments. For this sub-study assessing the delta for hs-cTnT, there were 1314 results obtained from 41 different analyzers (Roche Diagnostics, Rotkreuz, Switzerland: 9 e411, 4 e601, 14 e602, 14 e801 analyzers). The established target hs-cTnT values for the three levels were 6.2 ng/L, 8.5 ng/L, and 11.7 ng/L, respectively [18]. Given that hs-cTn results and their deltas are usually interpreted as whole numbers in clinical practice [28], these target levels were rounded off to 6 ng/L, 9 ng/L, and 12 ng/L and the corresponding delta values between these target levels was categorized as “low-delta” (i.e., level 2 minus level 1; 9 ng/L − 6 ng/L = 3 ng/L) versus “high-delta” (level 3 minus level 1; 12 ng/L − 6 ng/L = 6 ng/L). Previous data has indicated that rounding versus not rounding hs-cTn values close to the limit of the detection does not significantly affect diagnostic estimates for those patients designated as low-risk (i.e., rule-out arm), but does impact the proportion of patients assigned as low-risk [29]. As our goal was to mirror clinical practice, coupled with the fact that the delta cutoffs are reported to whole numbers (i.e., no decimal place), we elected to round all hs-cTn values prior to performing subtraction to derive the delta value. For each analyzer over the 12 months the actual results obtained were rounded to the nearest whole number and the delta calculated by subtraction (i.e., level 2 − level 1 = “measured @ low-delta”; level 3 − level 1 = “measured @ high-delta”).

Based on the different deltas as incorporated in the ESC, High-STEACS, and 3C pathways (see Table 1), we then classified each of the repeated measurements as follows: a correct classification at low-delta of 3 ng/L would be observe for ESC 0/1 h, low-risk for ESC 0/2 h, high-risk for High-STEACS, and low-risk for 3C. Correct classification with the assigned high-delta of 6 ng/L would be high-risk for ESC 0/1 h, observe for ESC 0/2 h, and high-risk for both the High-STEACS and 3C (see Table 1). The number of measured deltas per pathway that yielded the correct classification was then divided by the total number of deltas obtained to derive the percentage of correct classification.

We calculated the percentages with 95% confidence intervals [95% CI; Exact (Clopper-Pearson)] for correct classification for each pathway. We also performed non-parametric analyses (i.e., median, interquartile range; IQR, etc.) with normality (Shapiro–Wilk test) and histograms also derived for the measured deltas and for the difference between the measured and assigned deltas (Analyse-it and StatsDirect Statistical Software used for the data analysis), with *p*-value < 0.05 considered significant.

The parent study was approved at all sites by the institutional ethics review boards [27]. As this substudy was a quality assurance study assessing laboratory test reproducibility, and was not an interventional study, it did not require additional research ethics board approval [18,25,26]. Further information on the availability of materials or data availability is available upon request.

## 3. Results

**Figure 1:**
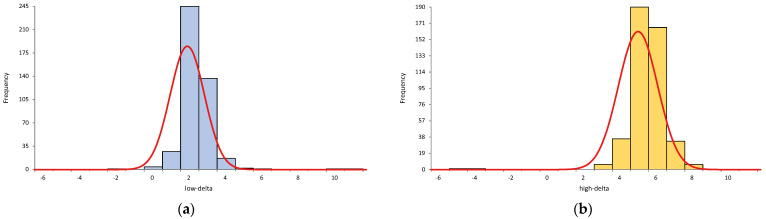
Histograms with an overlie of normal distribution curves for the measured deltas: (**a**) The measured low-deltas (n = 436) with the assigned value of 3 ng/L; (**b**) The measured high-deltas (n = 439) with the assigned value of 6 ng/L.

From 1314 hs-cTnT measurements, only one result yielded a value of <3 ng/L and was assigned a value of 3 ng/L to allow the calculation of a delta. The measured delta values at both the low- and high-delta (n = 875) were not normally distributed (*p* < 0.001, Shapiro–Wilk test) with the median (IQR), for the measured low-delta being 2 ng/L (2 to 3) and high-delta being 5 ng/L (5 to 6) (Figure 1). The relative frequency of the measured deltas at the assigned delta values was 31% for the low-delta (correct assignment was 3 ng/L) and 38% for the high-delta (correct assignment was 6 ng/L).

The median (IQR) differences between the measured and assigned deltas was −1 ng/L (−1 to 0) for both the low-delta and high-delta. The range of differences between the measured and the assigned deltas were −5 ng/L to 8 ng/L for the low-delta and −11 ng/L to 2 ng/L for the high-delta.

The percent correct classification for the pathways for the measured low-deltas and high-deltas ranged from 35.3% to 99.5%. For the low-delta with the correct classification being 3 ng/L, both the ESC 0/2 h and 3C classifications would be low-risk, while the classification for the ESC 0/1 h would be observe and the High-STEACS would be high-risk. Approximately one third of the measured deltas matched the correct classification for the ESC 0/1 h and High-STEACS pathways (Table 2). This contrasts with both the ESC 0/2 h and 3C pathways, where the measured deltas yielded 95% of the correct classification. The incorrect low-risk classification for the High-STEACS and ESC 0/1 h pathways was the same at 63.8% (95% CI: 59.0 to 68.3).

At the high-delta, only the ESC 0/2 h pathway classification was different (i.e., classification was observe), while in the three other pathways the classification was the same (i.e., high-risk). The lowest percent correct classification on the measured deltas was the ESC 0/1 h pathway at 90.0% (95% CI: 86.8 to 92.6) with the highest percent correct classification being the High-STEACS pathway at 99.5% (95% CI: 98.4. to 99.9) (Table 3). Both the ESC 0/2 h and 3C pathways measured deltas yielded 98.2% (95% CI: 96.4 to 99.2) correct classification. The incorrect low-risk classification for High-STEACS and the ESC 0/1 h pathways was the same at 0.5% (95% CI: 0.06 to 1.6) and 1.8% (95% CI: 0.8 to 3.6) for the ESC 0/2 h and 3C pathways

## 4. Discussion

The precise measurements for cardiac troponin at very low concentrations, well below the URL (usually below 10 ng/L), using high-sensitivity assays have led to the development of multiple pathways that can be used for early decision-making in the emergency setting for patients with possible MI [3,4,5,6,7,8]. There has been substantial focus on cTn concentration cutoff to provide an early rule-out as compared to a high concentration cutoff to rule-in [24,26,30,31,32]. In part, modeling data, even with non-hs-cTn assays have indicated that variation at high concentrations at and above the URL does not lead to misclassifications [30,32,33]. However, most of the published pathways use absolute deltas and, of these, the majority are single digit deltas to further classify patients as low-risk, intermediate (observe), or high-risk. The impact of analytical variation around these deltas on risk misclassification is largely unknown and complex to determine [4].

Our study has assessed the variation in a low-delta (3 ng/L) versus a high-delta (6 ng/L) with the hs-cTnT assay and its impact on correct classification using four distinct published cardiac troponin-based pathways (ESC 0/1 h, ESC 0/2 h, High-STEACS, 3C). Three major findings are evident. First, the potential for incorrect classification appears to be substantial when applying the low-delta, with nearly two thirds being incorrect with the ESC 0/1 h and High-STEACS pathways. Second, more correct classification occurs when using the high-delta with at least 90% correct classification being evident across all four pathways. Third, the ESC 0/2 h and 3C pathways were least vulnerable to misclassification when considering both deltas; perhaps in part due to a higher delta to classify patients as low-risk (i.e., <4 ng/L or ≤3 ng/L are equivalent deltas and identify changes of 3 ng/L or lower as low-risk). These data highlight that there is substantial variation using low-deltas in terms of patient risk classification per pathway applied and that the reproducibility on obtaining the correct delta also varies for each of the pathways. Accordingly, despite the fact that EDs are often extremely busy, clinicians need to understand how important it is to assess other variables such as the time of pain onset and other clinical assessments to augment the use of delta change criteria for risk-stratification.

The strengths of the present analysis include the multicenter setting, the use of different analyzers, the longitudinal nature of testing that captures variation across different reagent lots, and the fact that the measured and assigned deltas are clinically relevant for hs-cTnT and can be assessed by the four different pathways. Limitations include that only hs-cTnT was evaluated, and no hs-cTnI assays were included. Also, as laboratories were instructed and reminded on a monthly basis to analyze these three samples, it is most likely that all three samples were run one after the other, which does not reflect the real world setting for samples coming from the emergency setting and where variation may be higher as the measurements are hour(s) apart [34]. Additionally, whole numbers rather than numbers with decimal points were used in deriving the delta in this study. The latter approach on using whole numbers is further supported by a study of over 10,000 quality control measurements where the standard deviation at concentrations below and around 10 ng/L was found to be approximately ±1 ng/L [35].

## 5. Conclusions

From a clinical practice standpoint, most clinicians do not interpret deltas in isolation and often take a Bayesian approach, combining the laboratory data with the clinical story and history, the electrocardiogram, and a sense of pre-test probability for MI. That makes our study findings even more helpful, as they highlight which pathways can quietly support decision-making without steering clinicians wrong when considering test result variation. Some clinicians may be more likely to lean on pathways that align with the real-world uncertainty of deltas, as demonstrated in this paper. Analytical variation will impact risk classification for MI when using hs-cTn deltas alone, according to the pathways. The 3C and ESC 0/2 h pathways demonstrated <5% misclassification due to analytic variation when using both low and high deltas for hs-cTnT in this dataset. Given that hs-cTnI assays are widely used globally, and analytical performance characteristics differ, it is imperative that similar studies for all hs-cTnI assays are performed. These further studies with different hs-cTnI assays at concentrations below 10 ng/L and below the 99th percentile are warranted to confirm our study’s findings on the delta variability obtained with the fifth-generation hs-cTnT assay.

## 6. Patents

McMaster University has filed the following patent, “QUALITY CONTROL MATERIALS FOR CARDIAC TROPONIN TESTING”, with P.K. and L.C. being listed as inventors.

## Figures and Tables

**Table 1:** The deltas (i.e., change in concentration between two blood draws) for the ESC, High-STEACS, and 3C pathways with designated risk classification for an initial sample below the 99th percentile for the hs-cTnT assay.

Risk	ESC 0/1 h [ng/L]	ESC 0/2 h [ng/L]	High-STEACS [ng/L]	3C [ng/L]
Low-risk	<3	<4	<3	≤3
Observe	3 to 4	4 to 9	Not applicable ^1^	Not applicable ^1^
High-risk	≥5	≥10	≥3	>3

^1^ There is no observe (intermediate) zone using the delta for High-STEACS [22] and for the 3C pathway if the first concentration is <10 ng/L (i.e., level 1 target = 6 ng/L in this study) [8].

**Table 2:** The percent correct classification for each pathway with an assigned low-delta of 3 ng/L.

Pathway	Classification	Low-Delta Correct (95% CI)
ESC 0/1 h	observe	35.3%
	delta = 3 to 4 ng/L	(30.8 to 40.0)
ESC 0/2 h	low-risk	95.2%
	delta < 4 ng/L	(92.7 to 97.0)
High-STEACS	high-risk	36.2%
	delta ≥ 3 ng/L	(31.7 to 40.9)
3C	low-risk	95.2%
	delta ≤ 3 ng/L	(92.7 to 97.0)

**Table 3:** The percent correct classification for each pathway with an assigned high-delta of 6 ng/L.

Pathway	Classification	High-Delta Correct (95% CI)
ESC 0/1 h	high-risk	90.0%
	delta ≥ 5 ng/L	(86.8 to 92.6)
ESC 0/2 h	observe	98.2%
	delta 4 to 9 ng/L	(96.4 to 99.2)
High-STEACS	high-risk	99.5%
	≥ 3 ng/L	(98.4. to 99.9)
3C	high-risk	98.2%
	> 3 ng/L	(96.4 to 99.2)

## Data Availability

Please contact the corresponding author regarding further information on data availability.

## Abbreviations

The following abbreviations are used in this manuscript:

MI	Myocardial Infarction
hs-cTn	High-Sensitivity Cardiac Troponin
ESC	European Society of Cardiology
3C	Common Change Criteria
